# Long-Term Inhibition of Notch in A-375 Melanoma Cells Enhances Tumor Growth Through the Enhancement of *AXIN1, CSNK2A3*, and *CEBPA2* as Intermediate Genes in Wnt and Notch Pathways

**DOI:** 10.3389/fonc.2020.00531

**Published:** 2020-06-30

**Authors:** Faezeh Keyghobadi, Maryam Mehdipour, Vahab Nekoukar, Javad Firouzi, Abolfazl Kheimeh, Fatemeh Nobakht Lahrood, Vajihe Azimian Zavareh, Masoumeh Azimi, Mahsa Mohammadi, Niloofar Sodeifi, Marzieh Ebrahimi

**Affiliations:** ^1^Department of Developmental Biology, University of Science and Culture, ACECR, Tehran, Iran; ^2^Department of Stem Cells and Developmental Biology, Cell Science Research Center, Royan Institute for Stem Cell Biology and Technology, ACECR, Tehran, Iran; ^3^School of Electrical Engineering, Shahid Rajaee Teacher Training University, Tehran, Iran; ^4^Animal Core Facility, Reproductive Biomedicine Research Center, Royan Institute for Biotechnology, ACECR, Tehran, Iran; ^5^Department of Pathology, Reproductive Biomedicine Research Center, Royan Institute for Reproductive Biomedicine, ACECR, Tehran, Iran

**Keywords:** DAPT, Notch signaling pathway, melanoma, mathematical model, xenograft model

## Abstract

Notch suppression by gamma-secretase inhibitors is a valid approach against melanoma. However, most of studies have evaluated the short-term effect of DAPT on tumor cells or even cancer stem cells. In the present study, we surveyed the short-term and long-term effects of DAPT on the stem cell properties of A375 and NA8 as melanoma cell lines. The effects of DAPT were tested both *in vitro* and *in vivo* using xenograft models. In A375 with B-raf mutation, DAPT decreased the level of *NOTCH1, NOTH2*, and *HES1* as downstream genes of the Notch pathway. This was accompanied by enhanced apoptosis after 24 h treatment, arrest in the G_2−_M phase, and impaired ability of colony and melanosphere formation at the short term. Moreover, tumor growth also reduced during 13 days of treatment. However, long-term treatment of DAPT promoted tumor growth in the xenograft model and enhanced the number and size of colonies and spheroids *in vitro*. The gene expression studies confirmed the up-regulation of Wnt and Notch downstream genes as well as *AXIN1, CSNK2A3*, and *CEBPA2* following the removal of Notch inhibitor *in vitro* and in the xenograft model. Moreover, the Gompertz-based mathematical model determined a new drug resistance term in the present study. Our data supported that the long-term and not short-term inhibition of Notch by DAPT may enhance tumor growth and motility through up-regulation of *AXIN1, CSNK2A3*, and *CEBPA2* genes in B-raf mutated A375 cells.

## Introduction

Different signaling pathways can act as tumor amplifiers in melanoma such as Notch, Wnt, and Shh signaling pathways (1–5). Melanoma has been determined by its medical treatment failure, tumor recurrence, and rapid metastasis to distant organs (6). It has been reported that melanoma cells have the ability to revolve into a more embryonic or even stem cell type and express some stem cell-related markers including CD133, Nestin, CD166, and ABCB5 (7–9). Because of this, they are more invasive and resistant to certain anticancer treatments, and no single therapy is reliable in the treatment of melanoma. Therefore, a number of antitumor agents are developed against certain signaling pathways such as EGFR, AKT, and BRAF (10). Nevertheless, targeted tumor therapies can be restricted by the emergence of resistant tumor cells to BRAF inhibitors (11) and EGFR inhibitors (12).

Notch is an important developmental pathway in melanoma, and its alteration has been documented to induce the transition of melanocyte to melanoma cells, the promotion of angiogenesis, tumor progression, and invasion (13–17). Several different methods for Notch blockade have been attempted, including gamma-secretase inhibitors (GSIs) (16, 18–23), monoclonal antibodies (18), and small inhibitory molecules, which directly affect the transcriptional complex (18, 24, 25).

It has also been determined that treatment of melanoma cells with Notch inhibitors with GSIs can result in tumor regression (18, 26, 27). DAPT (*N*-[*N*-(3,5-difluorophenacetyl)-l-alanyl]-*S*-phenylglycine *t*-butyl ester) (28) is a well-known small molecule that has been reported to block Notch signaling in cancers (28) and has reduced cancer cell growth and invasive capacity of melanoma cells (16, 17, 29). Although most of the studies have evaluated the short-term effect of GSIs and the phase II clinical trials that explore the efficacy of GSIs in melanoma are actively recruiting or awaiting activation (30), but it is uncertain whether Notch signaling blockade alone and in the long term will be sufficient to prevent tumor growth as cancer adaptation is well-documented. Here, we report the short-term and long-term effects of Notch inhibition on stem cell ability as well as the tumor growth of malignant melanoma cells both *in vitro* and *in vivo* and evaluated the possible emergence of therapeutic resistance. Furthermore, by using mathematical models, on the basis of the tumor growth rate, we could estimate an optimal dosage of DAPT for supporting tumor regression in the xenograft mice and predict drug resistance at the proposed dose. Finally, the effect of DAPT in both short- and long-term administrations was assessed to evaluate the expression pattern of Notch and Wnt downstream genes, and their intermediate genes including *AXIN1, CSNK2A3*, and *CEBPA2* after removing the effect of DAPT.

## Materials and Methods

All procedures in the present study were performed in accordance with the relevant guidelines and regulations of the Royan Institute for Stem Cell Biology and Technology and approved by the Institutional Review Board and Ethics Committee of the Royan Institute, Tehran, Iran (IR.ACECR.ROYAN.REC.1396.28).

### Cell Culture

A375 human melanoma cell line originated from a culture of a lymph node metastasis of a melanoma patient (31), and NA8 (originated from the culture of malignant melanoma) was a gift from Dr. Giulio Spagnoli (University Hospital of Basel, Switzerland). Cells were cultured in complete Dulbecco's modified Eagle's medium (DMEM) high glucose from GIBCO [DEMEM, 10% fetal bovine serum (FBS), 1% nonessential amino acid, 1% l-glutamine, and 1% penicillin/streptomycin]. Cells were incubated at 37°C, 5% CO_2_.

### Short-Term and Long-Term Inhibition by DAPT

A375 cells were incubated with 15 μM of DAPT for 48 and 96 h as short -and long-term inhibition, respectively. The time was considered based on the changes in the percentage of apoptotic cells in treated cells (see Results section).

### Genomic Profiling of Cell Lines

To check the hotspot mutation of the *BRAF* gene at exon 15 and NRAS at exons 1 and 2, DNA was extracted from melanoma cell lines A375 and NA8, using a QIAamp DNA Mini Kit (Qiagen^®^ 51306, Hilden, Germany) according to the manufacturer's instructions. Primer pairs that targeted the human *BRAF* and *NRAS* genes were designed, and PCR was used to amplify the DNA region (Supplementary Table 1). The PCR products were submitted to conventional Sanger sequencing. Finally, samples were submitted to GenBank (BankIt) with accession numbers KY769663 and KY769668. Analysis and alignment of the data were performed by ChromasPro 2, CLC Sequence Viewer 6, and Gene Runner 5 software.

### MTS Assay

One thousand cells were seeded in 96-well plates and were incubated overnight at 37°C. Afterward, the media were changed with fresh media including different concentrations of DAPT (Tocris) (0, 1, and 15 μM for A375 cells and 0, 5, 10, 15, 30, and 60 μM for NA8 cells). Plates were incubated at 37°C for 24, 48, and 72 h. The media were removed, and 100 μl of MTS (Promega Co.) was added and incubated for an extra 3–4 h. The absorbance of the developed dye was measured at 560 nm with Thermo Scientific Elisa reader.

### Apoptosis Detection

A total of 5 × 10^5^ cells were washed with calcium buffer 1×. Annexin (Sigma) was added and incubated for 15 min at 4°C. Afterward, propidium iodide (PI) was added. Samples were acquisitioned by BD FACSCalibur in each step and analyzed by flowing software.

### Cell Cycle Analysis

A total of 5 × 10^6^ cells were washed with phosphate-buffered saline (PBS), and DNA staining method with PI was performed according to the Azimian-Zavareh et al. method (31).

### Colony and Sphere Formation Assay

Two hundred cells were seeded in six-well plates with complete DMEM, on the basis of our published protocol (32), to evaluate colony formation ability. To do sphere formation, 1 × 10^4^ cells were seeded in six-well plates coated with poly-Hema (1%) on the basis of the previously published protocol (32).

### Flow Cytometry Analysis

Nestin and Notch1 expression were evaluated by flow cytometry using specific antibodies against Nestin [1:100, fluorescein isothiocyanate (FITC) mouse anti-human, Santa Cruz] and Notch1 (1:100, FITC mouse anti-human, Santa Cruz). A total of 1 × 10^6^ cells were washed with PBS and fixed with paraformaldehyde 4%, permed with Triton X-100 (2%), and blocked with bovine serum albumin (BSA) 10% according to the Azimian-Zavareh et al. method (31).

To estimate c-Myc-positive (1:100, FITC mouse anti-human, Santa Cruz) and Ki-67-positive (1:100, FITC mouse anti-human BD) cells, tumors were treated with Dispase (Thermo Scientific), Collagenase type 1 (Abcam), and Collagenase type IV (Sigma) with a dose of 1 mg/ml of DMEM.

### Quantitative Gene Expression Using Real-Time Polymerase Chain Reaction

Total RNA was extracted using TRIzol reagent (Invitrogen). Then according to the previously described procedure (31), total RNA was extracted, and the expression of the genes mentioned in Table 1 was measured using SYBR Green Master Mix. *GAPDH* was used as an internal control.

**Table 1:** Primer sequences for real time PCR.

**Temperature Tm (°C)**	**Primer sequence**	**Gene name**
60°C	F: 5′CTC ATTTCCTGGTATGACAACGA 3′ R: 5′ CTT CCT CTT GTG CTC TTG ct 3′	GAPDH
60°C	F: 5′ AAAGAATCTTCACCTATGCC 3′ R: 5′ GAAGGAAGAGGAGAGACAGT 3′	Nanog
60°C	F: 5′ GTTCTTCATTCACTAAGGAAGGG 3′ R: 5′ CAA GAGCATCATTGAACTTCAC 3′	Oct-4
60°C	F: 5′ TCCAGGAACGGAAAATCAAG 3′ R: 5′ GCCTCCTCATCCCCTACTTC 3′	Nestin
60°C	F: 5′ GGCTAAGGTGTTTGGAGG 3′ R: 5′ TGTTGCTGGTGTAGACGG 3′	Hes1
60°C	F: 5′GAGGCGTGGCAGACTATGC 3′ R: 5′ CTTGTACTCCGTCAGCGTGA 3′	Notch1
60°C	R: 5′ GATCACCCGAATGGCTATGAAT 3′ R: 5′ GGGGTCACAGTTGTCAATGTT 3′	Notch2
60°C	F: 5′CATCTACACCGACAACTCCA 3′ R: 5′ ATGATCTGTTTGTTCTCCTCC 3′	CyclinD1
60°C	F: 5′GAGGCAACTATTTTAGACTGATTACTTT3′ R: 5′AGGTTAATGAGTGTCACAGACTTC3′	cateninβ
60°C	F:5′ CCAAGCGTGATCCTG AACC 3′ R: 5′ GCTGCTGCC GAGGAG TAG 3′	cMyc
60°C	F: 5′ GACCTGGGGTATGAGCCTGA 3′ R: 5′ GGCTTATCCCATCTTGGTCATC 3′	Axin1
60°C	F: 5′ GGTTCGTGACACAGGGTCTT 3′ R: 5′ CACATGTGGTGGAATGGGGA 3′	Csnk2A1
60°C	F: 5′ GTG GAA ACA TAG GGA CTT GG 3′ R: 5′ ATG ACA AAC AAG GCT GAG G 3′	CEBPA2

### Xenograft Mouse Model

All studies *in vivo* were done according to the guidelines for animal care established by the Royan Institute Animal Care Committee. A total of 2 × 10^6^ A375 cells were injected into the flanks of 6 to 8 week-old male nude mice (B6NU-M) purchased from Royan Research Institute (*n* = 31). After tumor development (about 1 week), mice were classified randomly in four groups. At day 0 after measuring the length and width of developed tumors, the treatment with DAPT was started. The control group received 10% dimethyl sulfoxide (DMSO) (the same concentration in DAPT solvent) (*n* = 6), the second group received intratumor (IT) injections of 12.97 mg/kg of DAPT (*n* = 6), the third group received intravenous (IV) injections of 259.6 mg/kg of DAPT (*n* = 6), and the fourth group (IT + IV) received 12.97 mg/kg of DAPT as IT and 259.6 mg/kg of DAPT as IV injections of (*n* = 13). DAPT treatment was performed when tumors reached to 4- to 6-mm diameter (100–150 mm^3^); the injection was done every 72 h up to 15 days. Tumor volume was assessed every day on the basis of the following formula:

$$\begin{array}{l} \text{Tumor volume}=\pi /6\times \text{Length}\times {\text{Width}}^{2} \end{array}$$

### Pathological Evaluation and H & E Staining

Mice tumors and lungs were harvested. Then the obtained samples were fixed in paraffin 10% and subjected to pathological evaluation and H&E staining.

### Mathematical Modeling

The proposed mathematical model describes the dynamics of tumor growth in the presence of DAPT. The model can be written based on the Gompertz equation (32) as follows:

(1) $$\begin{array}{l} ṅ\left(t\right)=\alpha n\left(t\right)\log\left(\frac{\theta }{n\left(t\right)}\right)-CK\left(u\left(t\right)\right)+R\left(u\left(t\right)\right) \end{array}$$

where *n* is tumor volume, ṅ is a derivative of *n, a* > *0* is the rate of tumor growth, and θ is the maximum tumor volume. *u*(*t*) is the schedule, the amount of DAPT injection is defined as *tCK*(*u*(*t*)), the cell-kill rate of DAPT, and *R*(*u*(*t*)) explain the drug resistance of tumor cells. *CK*(*u*(*t*)) and *R*(*u*(*t*)) are functions of *u*(*t*). Based on Equation (1), injection of DAPT increases the tumor size if *R*(*u*(*t*)) is bigger than *CK*(*u*(*t*)), and it decreases the tumor size if *CK*(*u*(*t*)) is bigger than *R*(*u*(*t*)). *CK*(*u*(*t*)) and *R*(*u*(*t*)) are calculated for both IV and IT injections. The parameters *a* and θ function as *CK*(*u*(*t*))*R*(*u*(*t*)) and should be estimated for every mice. *R*(*u*(*t*))−*CK*(*u*(*t*))is defined as the *killing factor* parameter, which determines if DAPT injection is useful or not for the specific animal.

### Statistical Analysis

The following data were presented as mean ± SD and analyzed by one-way ANOVA and *T*-test and *post-hoc* Bonferroni to assess differences among means using Prism version 6 software and SPSS. *P* < 0.05 was considered as significant. Experiments were repeated three times.

## Results

### DAPT Inhibits Cell Activity Transiently Through Short-Term Cell Cycle Arrest in A375 Melanoma Cells

**Figure 1:**
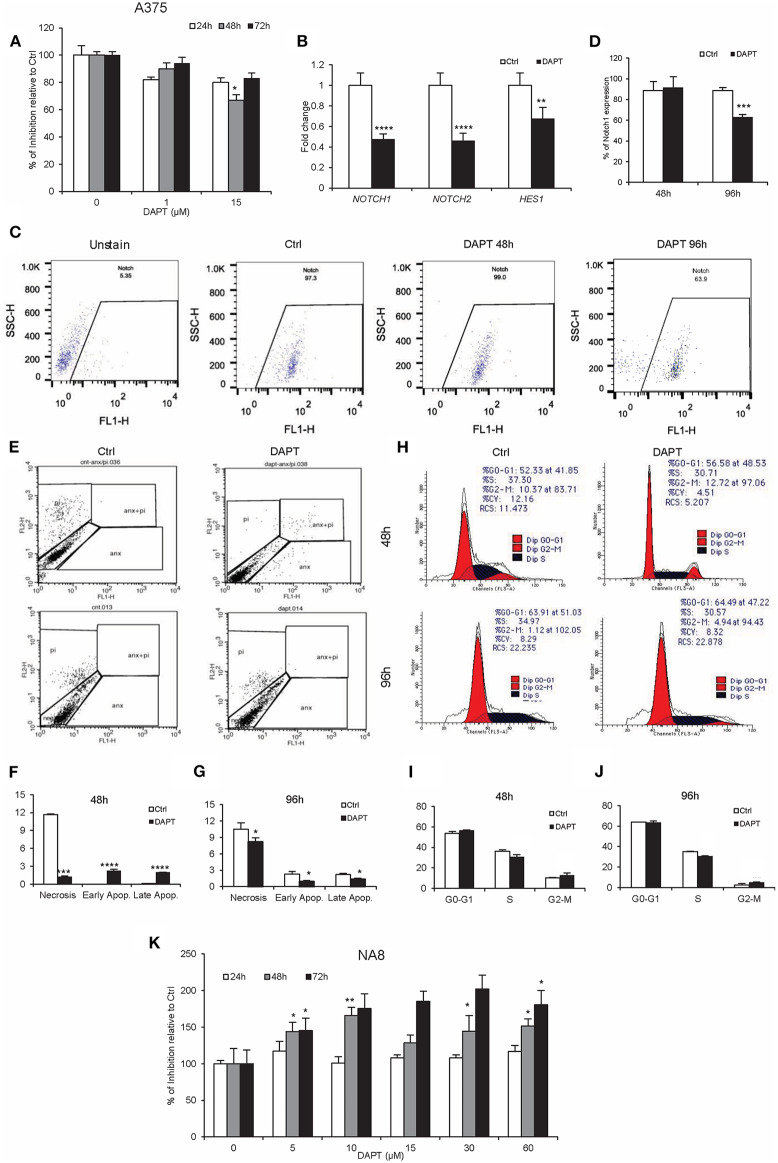
DAPT inhibits A375 cell activity temporarily. **(A)** MTS assay was done to show the inhibition of A375 activity with the dose of 15 μM of DAPT 48 h after treatment. **(B)** The treatment with DAPT resulted in the down-regulation of Notch downstream genes including *NOTCH1, NOTCH2*, and *HES1* after 48 h of treatment. The mRNA level was quantified by RT-PCR, and *GAPDH* was used as an internal control. **(C,D)** Flow cytometry analysis showed the reduction of Notch1 expression after 96 h of treatment. **(E–G)** Apoptosis assay was done to evaluate early and late apoptosis. Notch inhibition enhanced apoptosis in treated cells 48 h after treatment, but its rate was reduced 96 h after treatment. **(H–J)** Cell cycle analysis was evaluated by propidium iodide (PI) staining and analyzed by FACSCalibur. The results indicated a minor reduction in the S phase and an increase in the G2-M phase after treatment with DAPT. **(K)** NA8 cell line was resistant to the cytotoxic effect of DAPT, and its activity did not change after treatment. (All bars indicate mean ± SD in at least four independent tests. ^*^P < 0.05, ^**^P < 0.01, ^***^P < 0.001, and ^****^P < 0.0001).

The inhibitory effect of DAPT has been previously reported in many cancers including melanoma (33). Our results determined that 15 μM of DAPT partially inhibited A375 cells (metastatic cells), after 48 h of treatment (Figure 1A, *P* < 0.05), associated with the down-regulation of *NOTCH1, NOTCH2*, and *HES1* mRNA levels (Figure 1B, *P* < 0.01). Moreover, the percentage of *NOTCH1*-positive cells decreased in treated cells after 96 h of treatment (Figures 1C,D, *P* < 0.001). Early and late apoptosis increased 48 h after DAPT treatment but decreased significantly 96 h after the exposure of A375 with DAPT (Figures 1E–G, *P* < 0.05). Cell cycle analysis indicated a reduction in the S phase that was associated with an enhancement in the G_2_-M phase both 48 and 96 h after treatment, which was not significant (Figures 1H–J, *P* < 0.05). Unlike A375 cells, NA8 cells (malignant cells) showed an increase in cell division, in all doses and times after DAPT treatment (Figure 1K, *P* < 0.05). The sequencing results revealed that *BRAF* gene had a T to A transversion at nucleotide position (*BRAF* c.1799T>A) or amino acid position 600 just in A375, which resulted in an amino acid substitution from valine to glutamic acid at codon 600 (V600E) (Supplementary Figure 1A). Both cell lines did not have any mutation in NRAS gene (Supplementary Figure 1B).

### DAPT Reduces Stemness-Like Property in A375 Cells Transiently

**Figure 2:**
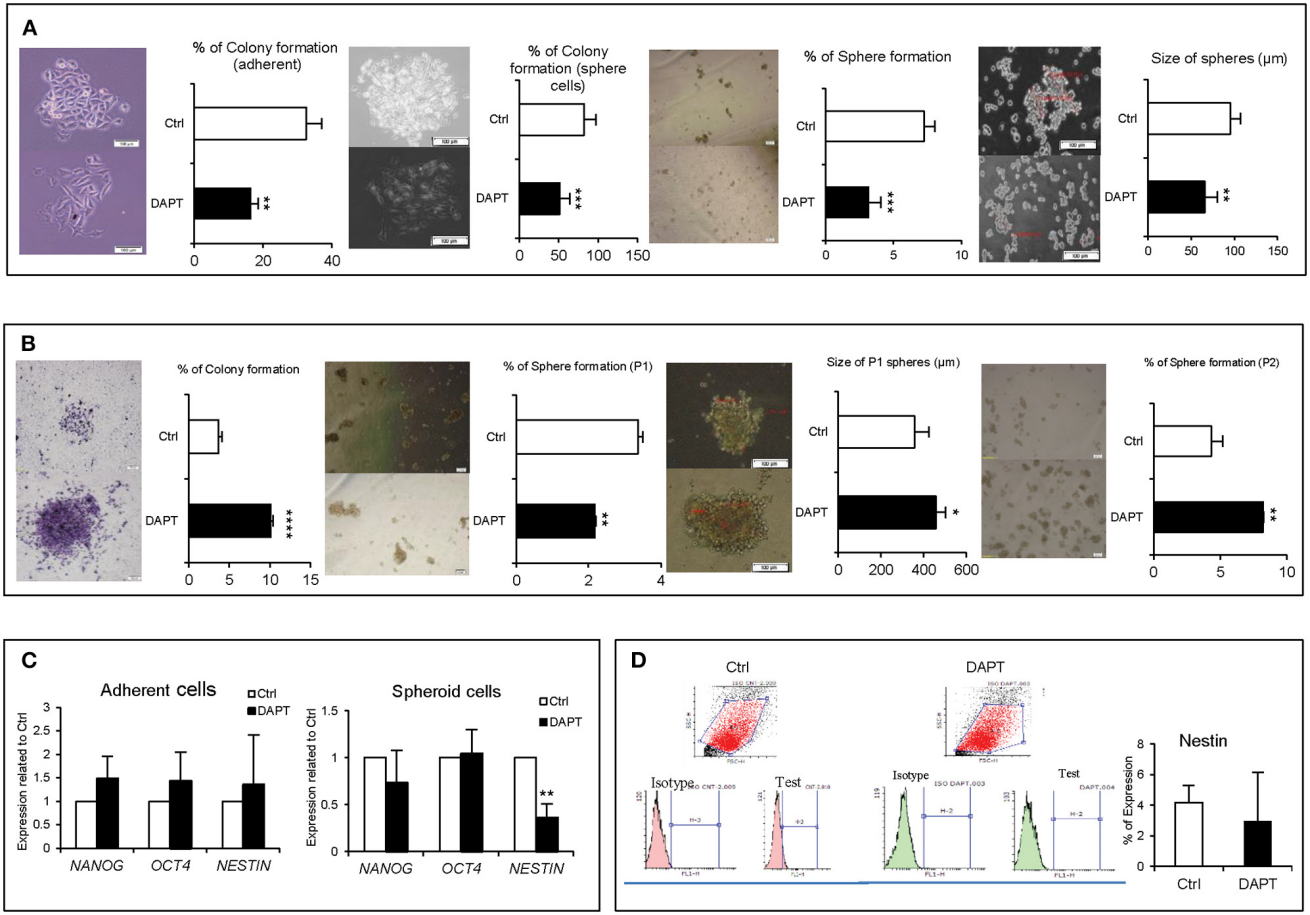
The effect of DAPT on A375 stemness properties. **(A)** Left to right: adherent and spheroid cells colony formation efficiency, as well as sphere formation ability decreased significantly after DAPT treatment. **(B)** Left to right: pre-treated cells (drug resistant cells) had an increased ability in their colony and sphere formation abilities. **(C)** q-RT-PCR analysis of stemness-related genes showed that 48 h treatment with DAPT enhanced the expression of *NANOG, OCT4*, and *NESTIN* in adherent culture. However, the changes were not significant (*P* > 0.05). It is unlikely that the expression of *NESTIN* significantly reduced at mRNA level in spheroid cells post treatment. **(D)** However, the changes were not significant at protein level (*n* = 3, mean ± SD, ^*^P < 0.05, ^**^P < 0.01, ^***^P < 0.001, ^****^P < 0.0001).

Our previous experiments indicated that DAPT was less effective on cell proliferation and apoptosis, but its effect on stemness properties such as colony and sphere formation and even stemness-related genes was unclear. Results showed that Notch inhibition in a short time dramatically reduced the colony and sphere formation abilities in both adherent and spheroid cells (Figure 2A). But long-term inhibition of Notch by DAPT in A375 cells increased the formation of larger colonies with a higher number (Figure 2B). Moreover, pre-treated cells with DAPT decreased the sphere number in sphere formation assay in passage 1, but the number and size of spheres significantly increased in the second sphere passages (Figure 2B). On the other hand, the mRNA level of *NANOG, OCT4*, and *NESTIN* increased in A375 cells treated with DAPT for 48 h. However, they were not significant (Figure 2C). It is unlikely that when spheroid cells were treated by DAPT, the expression of stemness-related genes such as *NESTIN* was significantly down-regulated (Figure 2C), but the protein level did not change after treatment (Figure 2D, *P* > 0.05). We conclude that DAPT can reduce the stemness-like properties of A375 cells in the short term, but in the long term, it has no effects on this function and may induce the drug resistance in treated cells, which should be confirmed by complementary tests.

### DAPT Reduces Sizes of Tumors Transiently in the Xenograft Mouse Model

**Figure 3:**
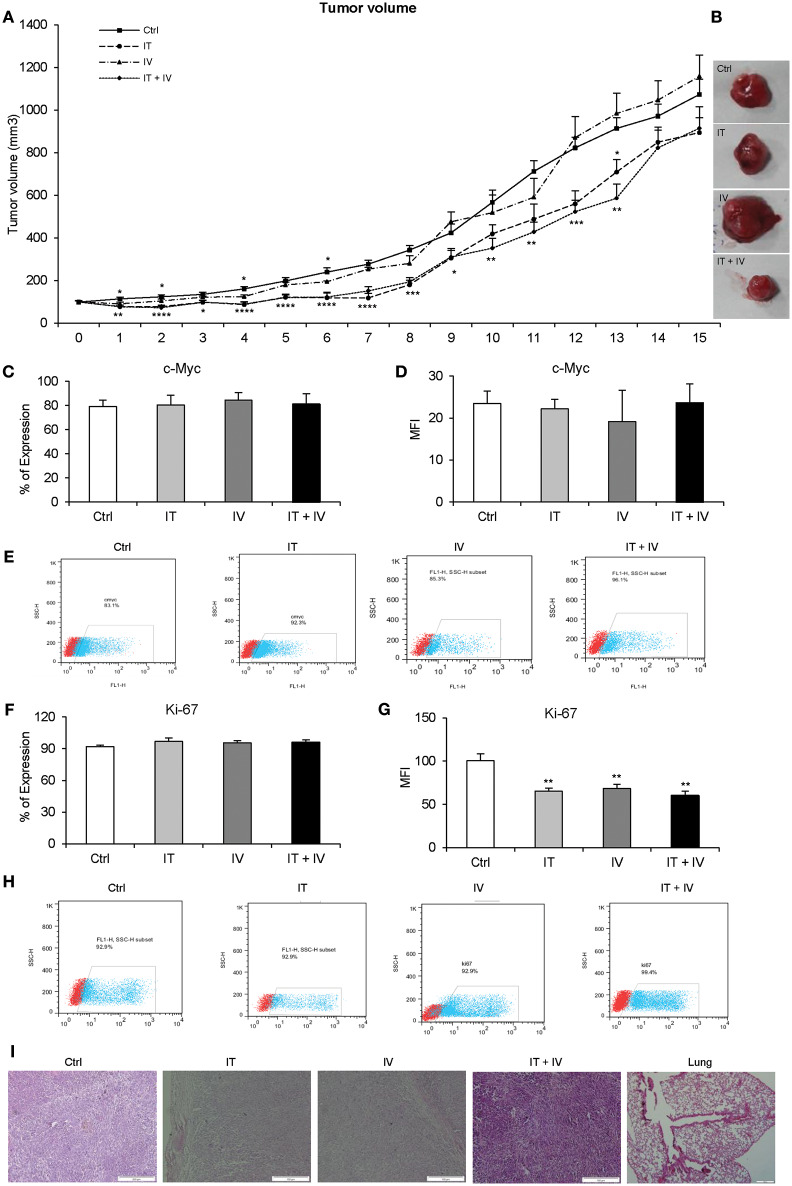
The effect of DAPT in melanoma mouse model. **(A)** A total of 2 × 10^6^ cells were injected into the flanks of 31 nude mice. The DAPT treatment was started when tumor size reached 4 to 6 mm diameters (100–150 mm^3^). Tumor-bearing mice were divided randomly into four groups: control, intratumor (IT) injections, intravenous (IV) injections, and IT + IV injections. The concentration for IT and IV injections was 12.97 and 259.6 mg/kg of DAPT, respectively, every 72 h (*n* = 6 for IT injection and IV injection, and *n* = 13 for IT + IV injection). For the control group, the mice received 10% dimethyl sulfoxide (DMSO) in phosphate-buffered saline (PBS) every 72 h (*n* = 6) for up to 15 days. The daily measurement of tumor volume indicated the decreasing rate of the tumor growth in the IV group for the first 6 days, but an increasing rate of the tumor growth in this group was observed up to the 15th day. The temporary reduction of tumor growth was observed in both IT and IT + IV groups during 13 days of treatment. However, size of tumors in all groups was not significant at day 15 after treatment. **(B)** The largest tumors in each group were pictured to show the morphology of tumors. **(C–H)** The level of Ki-67- and c-Myc-positive cells in each group (IT, IV, and IT+ IV injections) was assessed by flow cytometry 15 days after DAPT treatment. The results indicated no significant difference between groups. Red points show no staining, and the blue points show protein expression. **(I)** H&E staining for tumors in different groups. The lung tissue did not show any signs of metastasis (*n* = 6, mean ± SD, ^*^P < 0.05, ^**^P < 0.01, ^***^P < 0.001, ^****^P < 0.0001).

DAPT was injected by three different methods (IT, IV, and IT + IV) *in vivo*. As shown in Figures 3A,B, the growth of the tumors significantly decreased in the IV group until day 6 but eventually enhanced, which caused larger tumors than those in the control group at day 15. The pattern of tumor growth in IT and IT + IV groups revealed a significant decline until 13th day after treatment. However, 15 days after treatment, an intense increase was observed in tumor growth in both treated groups (Figures 3A,B). These results were also confirmed by histopathological evaluation (Table 2). Additionally, the percentage of positive cells and intensity of the c-Myc and Ki-67 in all groups were the same, and only the mean fluorescent intensity for Ki-67 decreased (Figures 3C–H, *P* > 0.01). This similar pattern of expression may be related to day of sampling, because we removed all tumors at day 15 with increasing tumor growth. If we had opportunity to sample tumors earlier (day 10), we might have observed differences in Ki-67 and c-Myc expression. These results of tumor growth were also confirmed by histopathological evaluation (Figure 3I, Table 2).

**Table 2:** Pathological Evaluations for tumors in each group.

**Name**	**Histology**	**Mitosis/hpf**	**Pleomorphism**	**Inflammation**	**Karyorrhexis**	**Ulcer**	**Desmoplasia**
Control	Nodular-type malignant melanoma	4–6	3	Moderate (lymphocytic)	30% Necrosis (10%)	Negative	Negative
IV	Nodular-type malignant melanoma	6	2–3	Mild (lymphocytic)	**10%**	Negative	Negative
IT + IV	Nodular-type malignant melanoma	4–6	3	Mild (lymphocytic)	**20%**	Negative	Negative

*Significant Values marked in bold P < 0.05*.

### Mathematical Model Evaluation and Optimal Therapy

**Figure 4:**
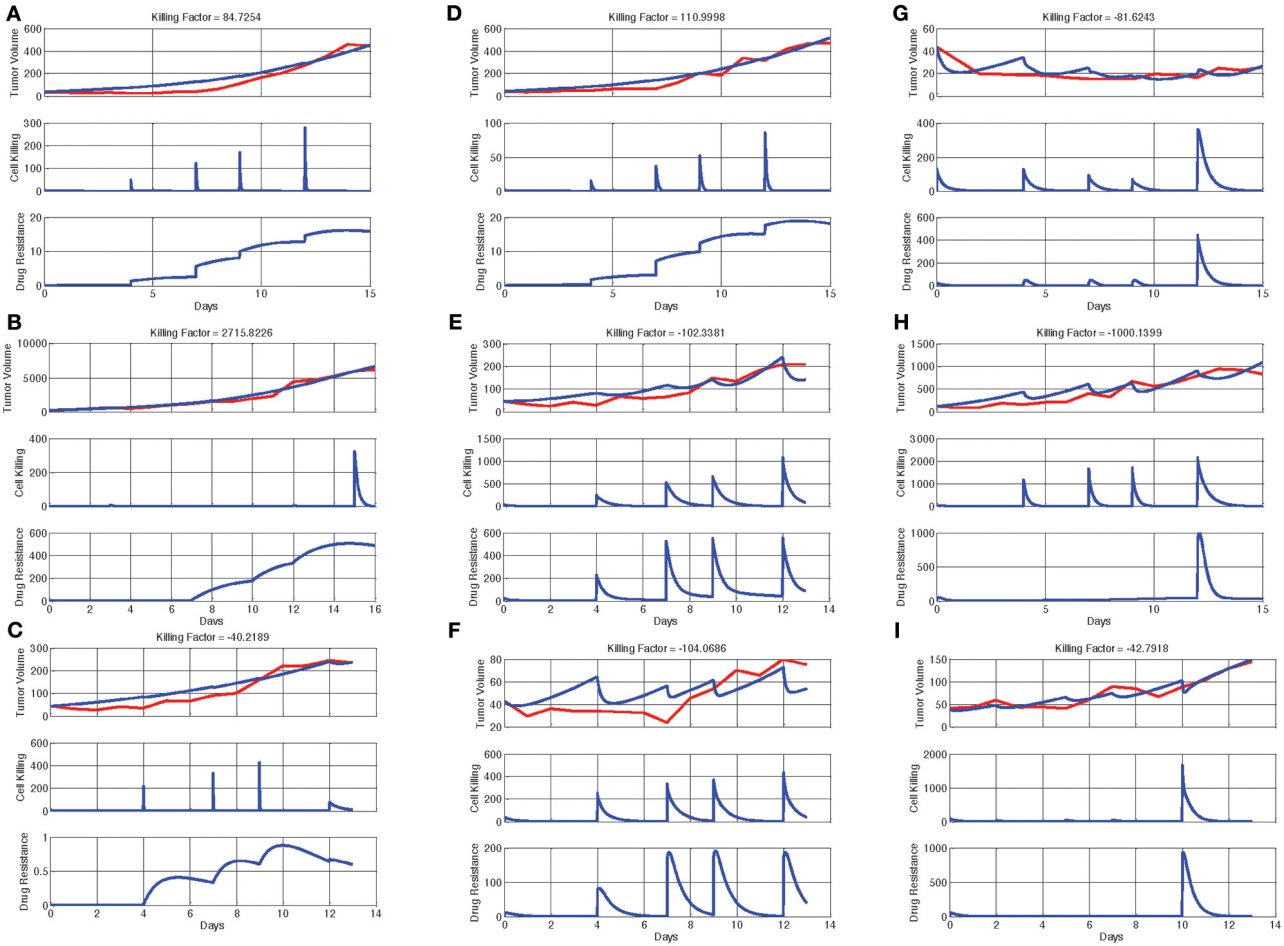
Output of the mathematical model (*n*(*t*)) and the measured tumor size, *CK*(*u*(*t*)), and *R*(*u*(*t*)) for four different animal models. Each graph has three parts: tumor volume, cell killing, and drug resistance. In the first part, the blue line shows real rate of tumor growth with DAPT treatment of control group in each mice, and the red line is the growth rate of DAPT treatment in each mice that were calculated with mathematical model. If the red and blue lines are more consistent, it means that the model is more similar to reality and more accurate. The total of these three parts provides killing factor: if the killing factor is negative, the drug was effective, and if the killing factor is positive, the treatment is harmful. **(A–D)** Animals with a positive killing factor were drug resistant. **(E,F)** Animals with a negative killing factor. These animals are remediable, and all of the injections were effective. **(G–I)** Animals with a negative killing factor. All of injections were not effective in these animals.

In the next step, we designed a mathematical model and identified model parameters for every animal by the generic algorithm. The mathematical model provides the opportunity to improve the therapy by considering killing factors, depicted on top of each plot. The output of the mathematical model (*n*) and the measured tumor size are shown for nine different animals in Figure 4 by red and blue lines, respectively. The second and third graphs of each plot indicate the functions of *CK*(*u*(*t*)) and *R*(*u*(*t*)), respectively. The results demonstrated that the model could explain the dynamics of the tumor size properly, because of the closeness of the estimated and measured tumor size. To determine DAPT effectiveness, the killing factor for all nine mice, shown in Table 3, was calculated. The positive value of the killing factors means that the specific injection was harmful. Based on the measured killing factor for the 2nd, 4th, 8th, 9th, and 10th animals, it was clear that DAPT was not a good choice for therapy. But the others show the negative killing factor, which indicated that DAPT controlled the tumor growth. Therefore, animal differences and the proportional dose are important factors in Notch inhibition. Thus, in order to find a systematic method for determining the optimal amount of DAPT for every injection, the optimal control theory was applied. The cost function *f*(*t*) was defined as follows:

(2) $$\begin{array}{l} f\left(t\right)=\int_{0}^{1}\left[n^{2}\left(\tau \right)+u_{IV}^{2}f\left(\tau \right)+u_{IT}^{2}f\left(t\right)\right]d\tau \end{array}$$

where *u*_*IV*_ and *u*_*IT*_ are related to the IV and IT injections, respectively. The optimal control tries to find a feasible dose of DAPT (*u*(*t*)) so that the cost function *f*(*t*) is minimized. Equation (2) shows that optimizing *f*(*t*) leads to minimizing the tumor size and the injection dose of DAPT, simultaneously. It is obvious that minimizing the injection amount of DAPT reduces the drug side effects (Supplementary Figure 2).

**Table 3:** Total killing factors for all mice calculated by mathematical method.

**Animal ID**	**Killing factor**	**Effectiveness**
1	−274	Remediable
2	**2,047**	**Irremediable**
3	−58	Remediable
4	**132**	**Irremediable**
5	−790	Remediable
6	−73	Remediable
7	−32	Remediable
8	**86**	**Irremediable**
9	**74**	**Irremediable**
10	**125**	**Irremediable**
11	−98	Remediable
12	−122	Remediable
13	−83	Remediable

*Significant Values marked in bold P < 0.05*.

To solve the optimal control, we assumed that the animal is injected with DAPT once every 3 days. The optimal schedule of every animal model is presented in Table 4 for the 15 days of therapy. Notably, the exclusive dose of DAPT was calculated for every animal. The advantage of this method is that drug resistance will be minimized. For the 8th, 9th, and 10th animals, the optimal therapy do not exist because IT killing factor is positive for them (see Table 4), which means IT injection of DAPT causes increase of the tumor growth rate.

**Table 4:** The optimal schedule of drug for every animal model.

**Animal ID**	***u*_*IV*_(*t*)**	***u*_*IT*_(*t*)**
0	X1-X1-X1-X1-X1-X1	X0-X0-X0-X0-X0-X3
1	X0-X0-X0-X0-X0-X0	X4-X4-X2-X2-X3-X4
2	X0-X1-X1-X1-X1-X1	X1-X3-X3-X3-X2-X1
6	X0-X0-X0-X0-X0-X0	X1-X1-X1-X1-X1-X1
7	X0-X0-X0-X0-X0-X0	X1-X1-X1-X1-X1-X1
8	X1-X1-X1-X1-X1-X0	X0-X0-X1-X0-X1-X0
9	X0-X0-X0-X0-X1-X1	X0-X1-X0-X1-X1-X0
10	X0-X1-X1-X1-X1-X1	X0-X0-X1-X1-X3-X4
11	X0-X1-X0-X1-X0-X1	X1-X0-X1-X0-X0-X0
12	X1-X1-X1-X1-X1-X1	X0-X0-X0-X0-X0-X0

### Is DAPT Suitable for Treatment of Melanoma Patients?

**Figure 5:**
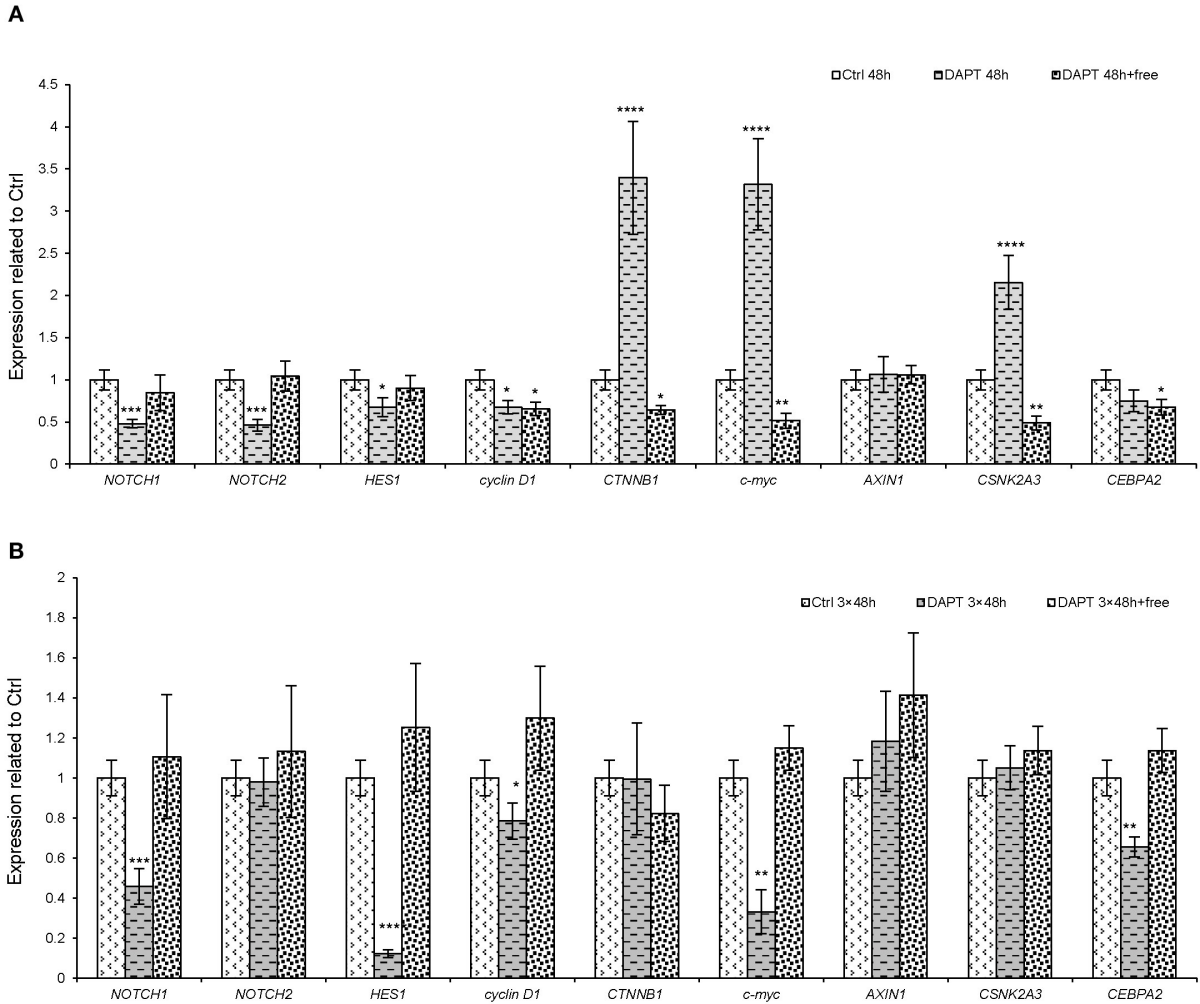
The expression of the downstream genes of both Notch and Wnt pathways and their intermediate genes after DAPT treatment. A375 cells were treated with DAPT in four groups. In the first group, the cells were treated with DAPT for 48 h, representing a short-term treatment. In the second group, initially, the cells, which were treated for 48 h, were washed and cultivated up to 1 week, without any treatment. In the third group, cells were treated for 6 days, representing a long-term treatment. Finally, in the fourth group, the cells that were treated for 6 days were washed and cultivated for an extra 1 week, without any treatment. The expression pattern of important genes in Notch and Wnt pathways and intermediate genes between these two pathways in groups 1 and 2 **(A)** and groups 3 and 4 **(B)** were assessed by qRT-PCR. In summary, it seems that the intermediate genes or Wnt pathway could retrieve the inhibitory effect of Notch pathway (*n* = 3, mean ± SD, ^*^P < 0.05, ^**^P < 0.01, ^***^P < 0.001, and ^****^P < 0.0001).

The results obtained from *in vitro* and *in vivo* experiments and the mathematical model revealed the differential responses of cells and animals to Notch inhibitors. Moreover, the effect of DAPT reversed after 13 days after Notch inhibition in the xenograft model. Therefore, several experiments were done to investigate the resistance mechanism of Notch inhibitors in melanoma cells, as both Notch and Wnt signaling pathways are interrelated and associated with tumorigenicity and metastasis in most cancer cells. Therefore, we selected several genes from the downstream of each of these pathways (*NOTCH1, NOTCH2*, and *HES1* for Notch pathway and *cyclin D1, c-myc*, and *CTNNB1* for Wnt signaling pathway). On the other hand, on the basis of the spectral partitioning model (data not shown), we found that *AXIN1, CSNK2A3*, and *CEBPA2* are intermediate genes between two pathways. Therefore, to seek the mechanism of resistance to Notch inhibitors, a set of experiments was done to clarify the gene roles. The results showed that the short-term treatment with DAPT (for 48 h) caused a reduction in Notch downstream genes (*NOTCH1, NOTCH2*, and *HES1*), but some Wnt pathway genes (*CTNNB1* and *c-myc*) and an intermediate gene (*CSNK2A3*) increased significantly (Figure 5A, *P* < 0.0001). The expression pattern of all genes returned almost to normal or even higher than normal when the short-term effect of DAPT was removed (after 48 h of treatment, cells were washed and cultured for a week). In contrast, the expression of CTNNB1, *c-myc*, and *CSNK2A3* declined (Figure 5A, *P* < 0.001). In the long-term treatment with DAPT (6 days of treatment that was equal to three cycles of therapy), the expression of *NOTCH1, HES1, cyclin D1, c-myc*, and *CEBPA2* was lower than that of the control group, but the expression of the other genes did not change. Interestingly, the expression of Wnt downstream genes, as well as the intermediate genes, in this case, was lower than normal (Figure 5B, *P* < 0.05). Finally, we found that the expression of Notch and Wnt downstream genes returned to normal when the effect of DAPT was removed in the long-term treatment (after 6 days' treatment with DAPT, cells were washed and cultured for a week) (Figure 5B); these data indicated that Notch inhibition alone is not sufficient for cell growth inhibition.

Based on the aforementioned results and the mathematical modeling, five tumors were selected to evaluate the pattern of Notch, Wnt, and intermediate genes (Table 5). It is noteworthy that on the 15th day of treatment, most tumors seemed to be developing resistance to the drug as they up-regulated *HES1, CTNNB1*, and all intermediate genes (*AXIN1, CSNK2A3*, and *CEBPA2*). Regarding this, it seems that DAPT failed to exert an effective therapeutic effect on the tumors.

**Table 5:** The expression of Notch and Wnt downstream genes and intermediate genes in five selected tumor 15th days post DAPT treatment.

**Gene/Tumor**	**Notch1**	**Notch2**	**Hes1**	**CyclinD1**	**β-catenin**	**c-Myc**	**Axin1**	**Csnk2A3**	**CEBPA2**
**Ctrl**	1	1	1	1	1	1	1	1	1
**3**	0.48 ± 0.07	0.54 ± 0.09	**2.77 ± 0.38**	0.67 ± 0.11	**1.22 ± 0.24**	**2.50 ± 0.52**	0.99 ± 0.19	2.37 ± 0.66	1.01± 0.09
**5**	0.26 ± 0.05	**1.52 ± 0.31**	**2.92 ± 0.45**	0.29 ± 0.04	**1.62 ± 0.31**	0.48 ± 0.11	**2.17 ± 0.51**	0.57 ± 0.10	**1.63 ± 0.32**
**7**	1.64 ± 0.21	**2.81 ± 0.55**	**2.80 ± 0.64**	1.28 ± 0.32	**1.88 ± 0.21**	0.54 ± 0.05	**5.31 ± 0.46**	**5.25 ± 0.89**	**2.11 ± 0.42**
**2**	0.90 ± 0.11	**7.95 ± 0.56**	**2.96 ± 0.33**	0.57 ± 0.08	**2.48 ± 0.32**	**2.82 ± 0.41**	**11.60 ± 1.09**	**1.27 ± 0.22**	**3.84 ± 0.44**
**9**	0.66 ± 0.13	**2.07 ± 0.33**	**2.86 ± 0.38**	0.62 ± 0.10	**1.74 ± 0.22**	**1.16 ± 0.36**	**3.39 ± 0.45**	**1.74 ± 0.25**	**3.85 ± 0.42**

*Significant Values marked in bold P < 0.05*.

## Discussion

Melanoma is identified as a heterogeneous cancer with a high degree of chemoresistance. Therefore, targeting resistant melanoma cells within tumors is necessary (26). Several reports have determined the role for aberrant Notch signaling in melanoma progression and have suggested that its inhibition may provide a valid therapeutic approach (16, 18).

DAPT is an effective and selective GSI with promising antitumor activities in several types of cancer, including melanoma (26, 34–36). However, its long-term effect is not clear. Our study indicated that DAPT affects melanoma cells in a cell-dependent manner and that it increases the cell activity of NA8 cells (as wild-type cell in *BRAF* gene) but suppresses A375 cells that contain *BRAF* mutation. We found that DAPT could increase apoptosis rate 48 h after treatment, which was reversed 96 h after treatment, which may be related to adaptation of treated cells with DAPT effect. Although DAPT-treated cells had changes in S phase and G2M of cell cycle, the differences were not significant. More importantly, it could suppress the tumor-initiating ability of melanoma cells in the short term, could partially decrease proliferation and colony formation, and could impair the formation of melanospheres. Similarly, several studies indicated the role of DAPT in the enhancement of apoptosis and cell cycle arrest in tongue carcinoma (34), colorectal cancer (35), and kidney carcinoma (37). Additionally, DAPT can reduce self-renewal and stemness in ovarian cancer stem-like cells (38) and breast cancer (39) and melanoma (40). However, our results determined that in the long-term treatment and after eliminating the effect of DAPT, melanoma cells became more aggressive with increase in both sphere and colony formation abilities. Similar to the *in vitro* experiment, the tumor size decreased in the treated group during the 13 days of treatment but suddenly increased and reached the size of the control group *in vivo*. It has been reported that the daily use of DAPT or the treatment of melanoma cells with high doses of DAPT significantly reduced the tumor growth rate (26) or tumorigenicity (36). On the contrary, a study in 2015 showed that DAPT could not reduce tumor growth (41) and that its monotherapy was reported to be incompetent (42). In this study, it seems that the reduction in tumor growth in the early stages of treatment in the first and second weeks is due to the reduction of tumor stem cell specificities. The obtained results lead to this notion that melanoma cells showed a differential response to DAPT on the basis of its dose and time exposure, both *in vitro* and *in vivo*. Furthermore, cells may bypass the Notch inhibition effect, using other mechanisms. We found that Notch blockade in short-term leads to the up-regulation of Wnt downstream genes (*CTNNB1* and *c-myc*) and *AXIN1, CSNK2A3*, and *CEBPA2*. Removing the blockade in treated cells results in the up-regulation of Notch-related genes and the down-regulation of Wnt genes. According to our results, we suggest that *CSNK2A3* may play an important role in providing balances between Notch and Wnt pathways. Moreover, the expression pattern of *AXIN, CSNK2A3, CEBPA2, HES1*, and *NOTCH2* was similar to that of most resistant tumors to DAPT, which had a similar growth rate with the control group. Axin (axis inhibition protein 1) is a scaffold protein involved in many signaling pathways, including Wnt, transforming growth factor-beta, MAP kinase pathways, and p53 activation cascades (43, 44). In addition, *AXIN1* is a mediator rather than an apoptosis marker (45) and is necessary for mediating the crosstalk between Wnt/β-catenin signaling cascades (46). Casein kinase 2 alpha 3 (*CSNK2A3*) encodes a protein that is highly similar to the casein kinase II alpha protein. Casein kinase II is a serine/threonine protein kinase (CK2) and is involved in cell growth, proliferation, apoptosis, and cell survival (47). CK2 functions as an oncogene in several types of cancer, and its up-regulation is correlated with low survival rates in most cases except for glioblastoma and renal cell carcinoma (48). CCAAT enhancer-binding protein alpha (CEBPA) is a transcription factor that is highly expressed in differentiated tissues. The overexpression of *CEBPA* is significantly correlated with the poor prognosis of ovarian cancer and hepatocellular carcinomas (HCCs), indicating its oncogenic role in these neoplasms. The down-regulation of this gene decreases colony formation and cell growth and increases the number of apoptotic cells (49). Moreover, the relationship between the Notch, Wnt, and PI3K downstream genes is previously identified (50). Recently, Norihiko Saito and colleagues have reported that GSI resistance results from a change in oncogene addiction, from NOTCH to constitutive AKT in glioblastoma, and that the combination of GSIs and PI3K inhibitors may reduce tumor growth in glioblastoma (51).

In addition, we introduced a mathematical model to determine the effectiveness of DAPT injection by calculating the killing factor. The mathematical model clearly showed that in most mice, DAPT was not a suitable choice for therapy. However, in several mice with negative killing factors, DAPT effectively controlled the tumor growth. Therefore, animal differences and dose proportions are important factors in notch inhibition.

In summary, notch inhibition may provide a novel approach for cancer therapy in melanoma, but some issues are of importance. (i) Notably, Notch inhibitors' effectiveness are cell and tumor dependent (52, 53); (ii) the dose and duration of Notch inhibitors (54, 55) and the gap between each therapy cycle are important factors. Although the high dose of DAPT is effective, the cytotoxicity may affect normal cells; (iii) resistance against Notch inhibitors may occur in the long term, leading to a more aggressive form of cancer with higher stem cell properties and metastasis; (iv) PI3K signaling, Wnt signaling pathways, and the other regulating networks may compensate the inhibition of Notch (41, 42, 50). Finally, the inhibition of Notch pathway leads to the overexpression of *AXIN1, CSNK2A3, CEBPA2, CTNNB1*, and *c-myc* genes and other unknown pathways that increase cell survival, metastasis, and drug resistance.

## Conclusion

Our data showed that DAPT blockade in melanoma leads to the up-regulation of *AXIN1, CSNK2A3, CEBPA2, CTNNB1*, and *c-my*c genes in tumors. All these events enhanced tumor growth, colonogenicity, and spheroid formation ability, which are associated with enhancement of cell motility in DAPT-resistant A375 cells. Our experimental and mathematical models indicated that the intratumor injection of DAPT is more effective than intravenous injection. However, the results should be confirmed by higher replications in long-term inhibition of NOTCH pathway.

## Data Availability Statement

The datasets generated for this study are available on request to the corresponding author.

## Ethics Statement

This study was carried out in accordance with the recommendations of animal guidelines, and approved by the ethical committee of Royan; IR.ACECR.ROYAN.REC.1396.28.

## Author Contributions

FK, MMe, JF, FN, and VA designed and performed experiments, analysed data, and co-wrote the paper. VN performed mathematical analyses. AK performed in animal modeling. MA and MMo performed flow cytometry analysis. NS contributed in pathological analysis. ME supervised the research and contributed in writing and revision. All authors read and approved paper for publication.

## Conflict of Interest

The authors declare that the research was conducted in the absence of any commercial or financial relationships that could be construed as a potential conflict of interest.

## Supplementary Material

Supplementary Figure 1 The sequencing of BRAF and NRAS mutation in A375 and NA8 cells. **(A)** The sequencing analysis showed that a T to A transversion at nucleotide position (BRAF c.1799T>A) or amino acid position 600 in A375 cells which resulted in an amino acid substitution from Valine to Glutamic Acid at codon 600 (V600E). **(B)** DNA sequence of the NRAS gene in exons 1 and 2 did not show any mutations neither in A375 nor the in D10 melanoma cell lines.

Supplementary Figure 2 Output of the mathematical model (n(t)) and the measured tumor size, CK(u(t)) and R(u(t)) for different animal models. Each graph has three parts: tumor volume, cell killing, and drug resistance. The blue line shows real rate of tumor growth with DAPT treatment to control group in each mice, and the red line is the growth rate of DAPT treatment in each mice that were calcuted with mathematical model. Whatever the lines of the red and blue are more consistent, it means that the model is more similar to reality and more accurate. The total of these 3 parts provide killing factor, which, if the killing factor was negative, the drug would be effective and if the killing factor was positive, treatment would be harmful. **(A–L)** Animals number 1 to 13.

Supplementary Table 1 The sequences of the primers used for sequencing.

## References

[1] Barat S Chen X Cuong Bui K Bozko P Götze J Christgen M. Gamma-Secretase Inhibitor IX (GSI) impairs concomitant activation of notch and wnt-beta-catenin pathways in CD44+ gastric cancer stem cells. Stem Cells Transl Med. (2017) 6:819–29. 10.1002/sctm.16-0335 28186678 PMC5442767

[2] Yahyanejad S King H Iglesias VS Granton PV Barbeau LM van Hoof SJ. NOTCH blockade combined with radiation therapy and temozolomide prolongs survival of orthotopic glioblastoma. Oncotarget. (2016) 7:41251. 10.18632/oncotarget.9275 27183910 PMC5173056

[3] Ponnurangam S Dandawate PR Dhar A Tawfik OW Parab RR Mishra PD. Quinomycin A targets Notch signaling pathway in pancreatic cancer stem cells. Oncotarget. (2016) 7:3217. 10.18632/oncotarget.6560 26673007 PMC4823101

[4] Nencini A Pratelli C Quinn JM Salerno M Tunici P De Robertis A. Structure–activity relationship and properties optimization of a series of Quinazoline-2, 4-diones as inhibitors of the canonical Wnt pathway. Eur J Med Chem. (2015) 95:526–45. 10.1016/j.ejmech.2015.03.055 25847770

[5] Sun M Zhang N Wang X Li Y Qi W Zhang H. Hedgehog pathway is involved in nitidine chloride induced inhibition of epithelial-mesenchymal transition and cancer stem cells-like properties in breast cancer cells. Cell Biosci. (2016) 6:44. 10.1186/s13578-016-0104-8 27313840 PMC4910241

[6] Flah1erty KT Hodi FS Fisher DE. From genes to drugs: targeted strategies for melanoma. Nat Rev Cancer. (2012) 12:349. 10.1038/nrc3218 22475929

[7] Brychtova S Fiuraskova M Brychta T Hirnak J. The role of intermedial filament nestin in malignant melanoma progression. Ceskoslovenska Patol. (2005) 41:143–5. 10.5306/wjco.v3.i3.32 16382989

[8] Fusi A Reichelt U Busse A Ochsenreither S Rietz A Maisel M. Expression of the stem cell markers nestin and CD133 on circulating melanoma cells. J Investig Dermatol. (2011) 131:487–94. 10.1038/jid.2010.285 20882037

[9] Frank NY Margaryan A Huang Y Schatton T Waaga-Gasser AM Gasser M. ABCB5-mediated doxorubicin transport and chemoresistance in human malignant melanoma. Cancer Res. (2005) 65:4320–33. 10.1158/0008-5472.CAN-04-3327 15899824

[10] Shtivelman E Davies MA Hwu P Yang J Lotem M Oren M. Pathways and therapeutic targets in melanoma. Oncotarget. (2014) 5:1701. 10.18632/oncotarget.1892 24743024 PMC4039128

[11] Manzano JL Layos L Bugés C de los Llanos Gil M Vila L Martínez-Balibrea E. Resistant mechanisms to BRAF inhibitors in melanoma. Ann Transl Med. (2016) 4:237. 10.21037/atm.2016.06.07 27429963 PMC4930524

[12] Huang L Fu L Mechanisms of resistance to EGFR tyrosine kinase inhibitors. Acta Pharm Sin B. (2015) 5:390–401. 10.1016/j.apsb.2015.07.001 26579470 PMC4629442

[13] King R Googe PB Weilbaecher KN Mihm MC Jr Fisher DE. Microphthalmia transcription factor expression in cutaneous benign, malignant melanocytic, and nonmelanocytic tumors. Am J Surg Pathol. (2001) 25:51–7. 10.1097/00000478-200101000-00005 11145251

[14] Garraway LA Widlund HR Rubin MA Getz G. Integrative genomic analyses identify MITF as a lineage survival oncogene amplified in malignant melanoma. Nature. (2005) 436:117. 10.1038/nature03664 16001072

[15] White RM Zon LI. Melanocytes in development, regeneration, and cancer. Cell Stem Cell. (2008) 3:242–52. 10.1016/j.stem.2008.08.005 18786412

[16] Balint K Xiao M Pinnix CC Soma A Veres I Juhasz I. Activation of Notch1 signaling is required for β-catenin–mediated human primary melanoma progression. J Clin Investig. (2005) 115:3166–76. 10.1172/JCI25001 16239965 PMC1257536

[17] Murtas D Piras F Minerba L Maxia C Ferreli C Demurtas P. Activated Notch1 expression is associated with angiogenesis in cutaneous melanoma. Clin Exp Med. (2015) 15:351–60. 10.1007/s10238-014-0300-y 25034654

[18] Takebe N Nguyen D Yang SX. Targeting notch signaling pathway in cancer: clinical development advances and challenges. Pharmacol Ther. (2014) 141:140–9. 10.1016/j.pharmthera.2013.09.005 24076266 PMC3982918

[19] Hoek K Rimm DL Williams KR Zhao H Ariyan S Lin A. Expression profiling reveals novel pathways in the transformation of melanocytes to melanomas. Cancer Res. (2004) 64:5270–82. 10.1158/0008-5472.CAN-04-0731 15289333

[20] Hsu MY Yang MH Schnegg CI Hwang S Ryu B Alani RM. Notch3 signaling-mediated melanoma–endothelial crosstalk regulates melanoma stem-like cell homeostasis and niche morphogenesis. Lab Investig. (2017) 97:725–36. 10.1038/labinvest.2017.1 28165469 PMC5446297

[21] Bagheri L Pellati A Rizzo P Aquila G Massari L De Mattei M. Notch pathway is active during osteogenic differentiation of human bone marrow mesenchymal stem cells induced by pulsed electromagnetic fields. J Tissue Eng Regen Med. (2018) 12:304–15. 10.1002/term.2455 28482141

[22] Tolcher AW Messersmith WA Mikulski SM Papadopoulos KP Kwak EL Gibbon DG Phase I study of RO4929097, a gamma secretase inhibitor of Notch signaling, in patients with refractory metastatic or locally advanced solid tumors. J Clin Oncol. (2012) 30:2348–53. 10.1200/JCO.2011.36.8282 22529266 PMC5950496

[23] Lee SM Moon J Redman BG Chidiac T Flaherty LE Zha Y. Phase 2 study of RO 4929097, a gamma-secretase inhibitor, in metastatic melanoma: SWOG 0933. Cancer. (2015) 121:432–40. 10.1002/cncr.29055 25250858 PMC4304973

[24] Pinnix CC Lee JT Liu ZJ McDaid R Balint K Beverly LJ. Active Notch1 confers a transformed phenotype to primary human melanocytes. Cancer Res. (2009) 69:5312–20. 10.1158/0008-5472.CAN-08-3767 19549918 PMC2755513

[25] Rehman M Gurrapu S Cagnoni G Capparuccia L Tamagnone L. Plexind1 is a novel transcriptional target and effector of notch signaling in cancer cells. PloS ONE. (2016) 11:e0164660. 10.1371/journal.pone.0164660 27749937 PMC5066946

[26] Huynh C Poliseno L Segura MF Medicherla R Haimovic A Menendez S. The novel gamma secretase inhibitor RO4929097 reduces the tumor initiating potential of melanoma. PloS ONE. (2011) 6:e25264. 10.1371/journal.pone.0025264 21980408 PMC3182998

[27] Zhu G Yi X Haferkamp S Hesbacher S Li C Goebeler M. Combination with γ-secretase inhibitor prolongs treatment efficacy of BRAF inhibitor in BRAF-mutated melanoma cells. Cancer Lett. (2016) 376:43–52. 10.1016/j.canlet.2016.03.028 27000992

[28] Olsauskas-Kuprys R Zlobin A Osipo C. Gamma secretase inhibitors of Notch signaling. Oncotargets Ther. (2013) 6:943. 10.2147/OTT.S33766 23901284 PMC3726525

[29] Vartanian A Gatsina G Grigorieva I Solomko E Dombrovsky V Baryshnikov A. The involvement of Notch signaling in melanoma vasculogenic mimicry. Clin Exp Med. (2013) 13:201–9. 10.1007/s10238-012-0190-9 22627943

[30] http://www.clinicaltrials.gov.

[31] Azimian-Zavareh V Hossein G Ebrahimi M Dehghani-Ghobadi Z. Wnt11 alters integrin and cadherin expression by ovarian cancer spheroids and inhibits tumorigenesis and metastasis. Exp Cell Res. (2018) 369:90–104. 10.1016/j.yexcr.2018.05.010 29753625

[32] Rahimi M Sharifi-Zarchi A Firouzi J Azimi M Zarghami N Alizadeh E. An integrated analysis to predict micro-RNAs targeting both stemness and metastasis in breast cancer stem cells. J Cell Mol Med. (2019) 23:2442–56. 10.1111/jcmm.14090 30710426 PMC6433858

[33] Nueda ML Naranjo AI Baladrón V Laborda J. The proteins DLK1 and DLK2 modulate NOTCH1-dependent proliferation and oncogenic potential of human SK-MEL-2 melanoma cells. Biochim Biophys Acta. (2014) 1843:2674–84. 10.1016/j.bbamcr.2014.07.015 25093684

[34] Grottkau BE Chen X-R Friedrich C Yang X-M Jing W Wu Y. DAPT enhances the apoptosis of human tongue carcinoma cells. Int J Oral Sci. (2009) 1:81–9. 10.4248/ijos.08025 20687300 PMC3735796

[35] Natarajan G Marimuthu S Perumal M Mani T Halagowder D. Colocalization of b-catenin with Notch intracellular domain in colon cancer: a possible role of Notch1 signaling in activation of CyclinD1-mediated cell proliferation. Mol Cell Biochem. (2014) 396:281–93. 10.1007/s11010-014-2163-7 25073953

[36] Bedogni B Warneke JA Nickoloff BJ Giaccia AJ Powell MB. Notch1 is an effector of Akt and hypoxia in melanoma development. J Clin Investig. (2008) 118:3660–70. 10.1172/JCI36157 18924608 PMC2567838

[37] Kerong W Lizhang I Yiweilin I Kai Y Yue C Inhibition of γ secretase induces G2/M arrest and triggers apoptosis in renal cell carcinoma. Oncol Lett. (2014) 8:55–61. 10.3892/ol.2014.2078 24959218 PMC4063651

[38] Jiang L-Y Zhang X-L Du P Zheng JH. γSecretase inhibitor, DAPT inhibits self-renewal and stemness maintenance of ovarian cancer stem-like cells *in vitro*. Chin J Cancer Res. (2011) 23:140–6. 10.1007/s11670-011-0140-1 23482909 PMC3587542

[39] Séveno C Loussouarn D Bréchet S Campone M Juin P Barillé-Nion S. g-Secretase inhibition promotes cell death, Noxa upregulation, and sensitization to BH3 mimetic ABT-737 in human breast cancer cells. Breast Cancer Res. (2012) 14:R96. 10.1186/bcr3214 22703841 PMC3446359

[40] Lin X Sun B Zhu D Zhao X Sun R Zhang Y. Notch4+ cancer stem like cells promote the metastatic and invasive ability of melanoma. Cancer Sci. (2016) 107:1079–91. 10.1111/cas.12978 27234159 PMC4982579

[41] Su F Zhu S Ruan J Muftuoglu Y Zhang L Yuan Q. Combination therapy of RY10–4 with the γsecretase inhibitor DAPT shows promise in treating HER2-amplified breast cancer. Oncotarget. (2015) 7:4142–54. 10.18632/oncotarget.6769 26716652 PMC4826195

[42] Jun Y Cuijuan Q Ting S Xin Z Zhiqiang Z Yong L. Combination treatment of PD98059 and DAPT in gastric cancer through induction of apoptosis and downregulation of WNT/β-catenin. Cancer Biol Ther. (2013) 9:833–9. 10.4161/cbt.25332 23792588 PMC3909552

[43] Lu Z Liu W Huang H He Y Han Y Rui Y. Protein encoded by the axinfu allele effectively down-regulates wnt signaling but exerts a dominant negative effect on c-Jun N-terminal kinase signaling. J Biol Chem. (2008) 283:13132–9. 10.1074/jbc.M710595200 18316368

[44] Chen Y Liang Z Fei E Chen Y Zhou X Fang W. Axin regulates dendritic spine morphogenesis through Cdc42-dependent signaling. PloS ONE. (2015) 10:e0133115. 10.1371/journal.pone.0133115 26204446 PMC4512687

[45] Biechele TL Kulikauskas RM Toroni RA Lucero OM Swift RD James RG. Wnt/β-catenin signaling and AXIN1 regulate apoptosis triggered by inhibition of the mutant kinase BRAFV600E in human melanoma. Sci Signal. (2012) 5:ra3. 10.1126/scisignal.2002274 22234612 PMC3297477

[46] Conrad WH Swift RD Biechele TL Kulikauskas RM Moon RT Chien AJ. Regulating the response to targeted MEK inhibition in melanoma: enhancing apoptosis in NRAS-and BRAF-mutant melanoma cells with Wnt/β-catenin activation. Cell Cycle. (2012) 11:3724–30. 10.4161/cc.21645 22895053 PMC3495814

[47] Ortega CE Seidner Y Dominguez I. Mining CK2 in cancer. PloS ONE. (2014) 9:e115609. 10.1371/journal.pone.0115609 25541719 PMC4277308

[48] Chua MM Lee M Dominguez I. Cancer-type dependent expression of CK2 transcripts. PloS ONE. (2017) 12:e0188854. 10.1371/journal.pone.0188854 29206231 PMC5714396

[49] Konopka B Szafron LM Kwiatkowska E Podgorska A Zolocinska A Pienkowska-Grela B. The significance of c. 690G> T polymorphism (rs34529039) and expression of the CEBPA gene in ovarian cancer outcome. Oncotarget. (2016) 7:67412. 10.18632/oncotarget.11822 27602952 PMC5341885

[50] Xiao W Chen X He M. Inhibition of the Jagged/Notch pathway inhibits retinoblastoma cell proliferation via suppressing the PI3K/Akt, Src, p38MAPK and Wnt/β catenin signaling pathways. Mol Med Rep. (2014) 10:453–8. 10.3892/mmr.2014.2213 24805975

[51] Saito N Hirai N Aoki K Suzuki R Fujita S Nakayama H. The oncogene addiction switch from NOTCH to PI3K requires simultaneous targeting of NOTCH and PI3K pathway inhibition in glioblastoma. Cancers. (2019) 11:121. 10.3390/cancers11010121 30669546 PMC6356490

[52] Panelos J Massi D. Emerging role of Notch signaling in epidermal differentiation and skin cancer. Cancer Biol Ther. (2009) 8:1986–93. 10.4161/cbt.8.21.9921 19783903

[53] Wang Z Li Y Ahmad A Azmi AS Banerjee S Kong D. Targeting Notch signaling pathway to overcome drug resistance for cancer therapy. Biochim Biophys Acta. (2010) 1806:258–67. 10.1016/j.bbcan.2010.06.001 20600632 PMC2955995

[54] Miele L Golde T Osborne B Notch signaling in cancer. Curr Mol Med. (2006) 6:905–18. 10.2174/156652406779010830 17168741

[55] Nueda ML Naranjo AI Baladrón V Laborda J. The proteins DLK1 and DLK2 modulate NOTCH1-dependent proliferation and oncogenic potential of human SK-MEL-2 melanoma cells. Biochim Biophys Acta. (2014) 1843:2674–84. 10.1016/j.bbamcr.2014.07.015 25093684

