# Supplementary figures and images for: Long-Term Inhibition of Notch in A-375 Melanoma Cells Enhances Tumor Growth Through the Enhancement of AXIN1, CSNK2A3, and CEBPA2 as Intermediate Genes in Wnt and Notch Pathways
Source: Front Oncol. 2020 Jun 30;10:531. doi: 10.3389/fonc.2020.00531 (PMC7338939; doi:10.3389/fonc.2020.00531)

A

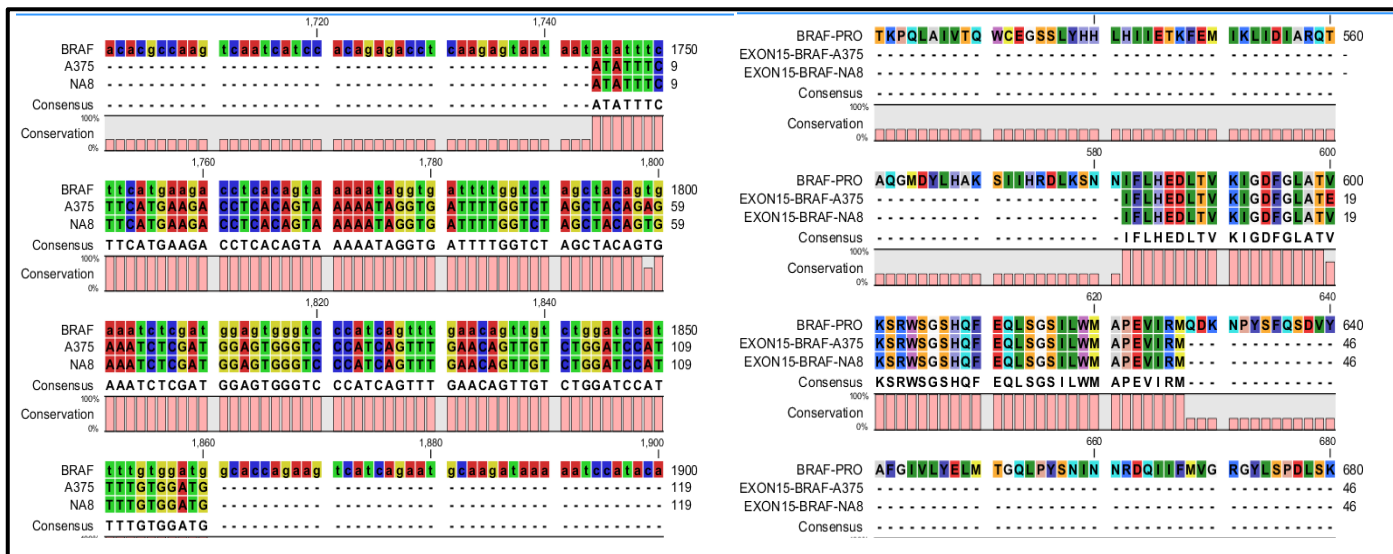

B

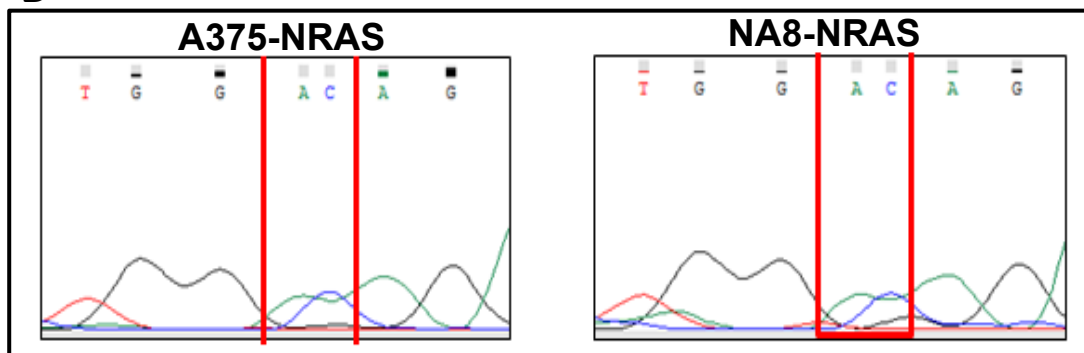

Supplement: Supplementary Figure 1 — The sequencing of BRAF and NRAS mutation in A375 and NA8 cells. (A) The sequencing analysis showed that a T to A transversion at nucleotide position (BRAF c.1799T>A) or amino acid position 600 in A375 cells which resulted in an amino acid substitution from Valine to Glutamic Acid at codon 600 (V600E). (B) DNA sequence of the NRAS gene in exons 1 and 2 did not show any mutations neither in A375 nor the in D10 melanoma cell lines. [file Data_Sheet_1.PDF]

A

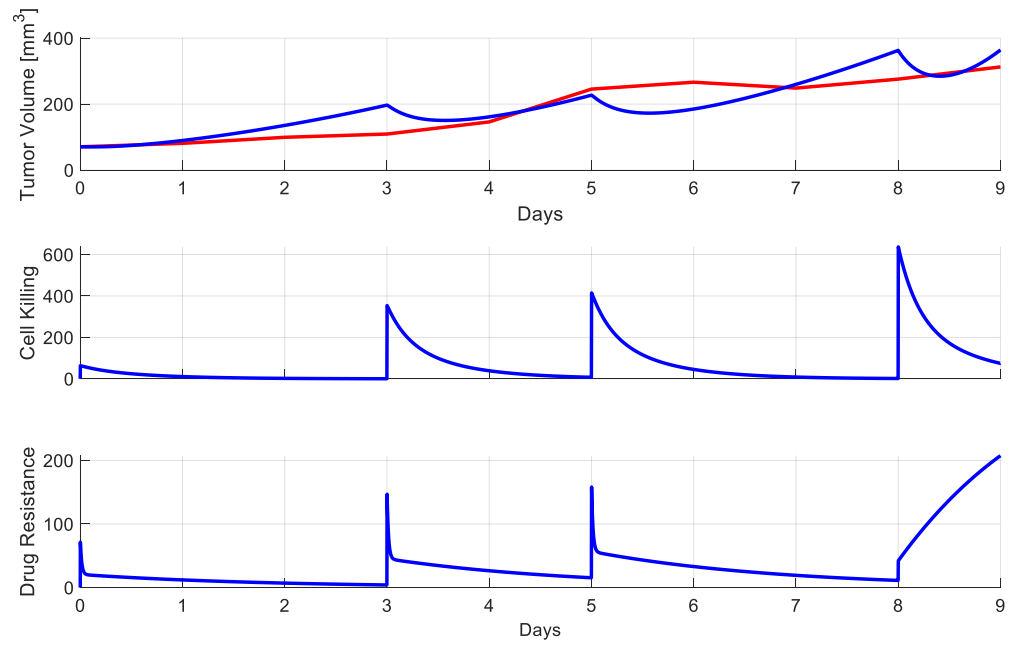

B

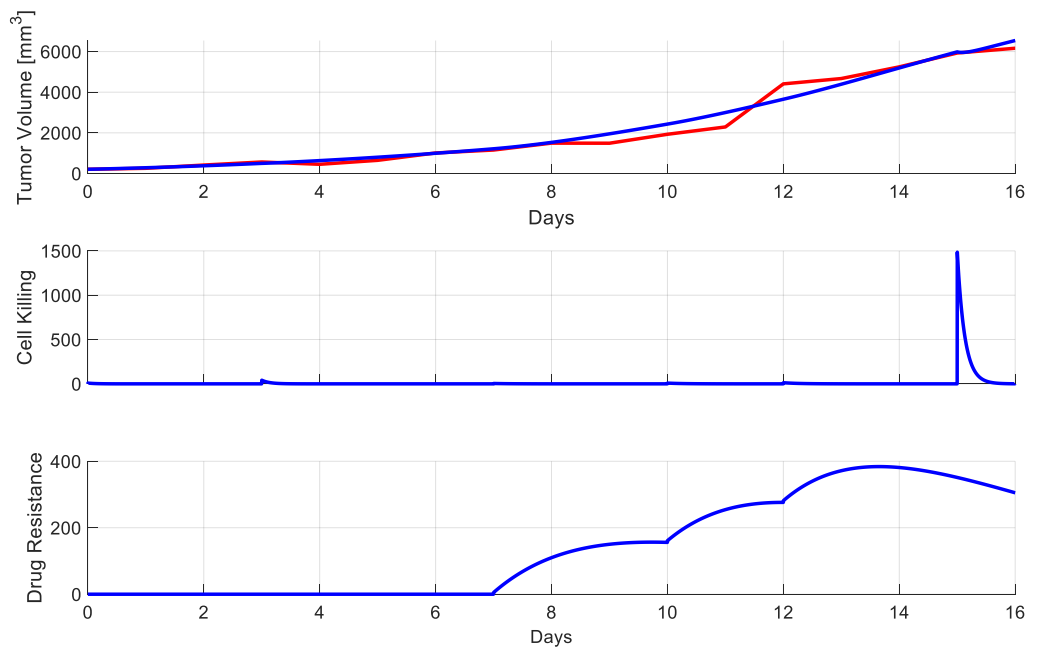

C

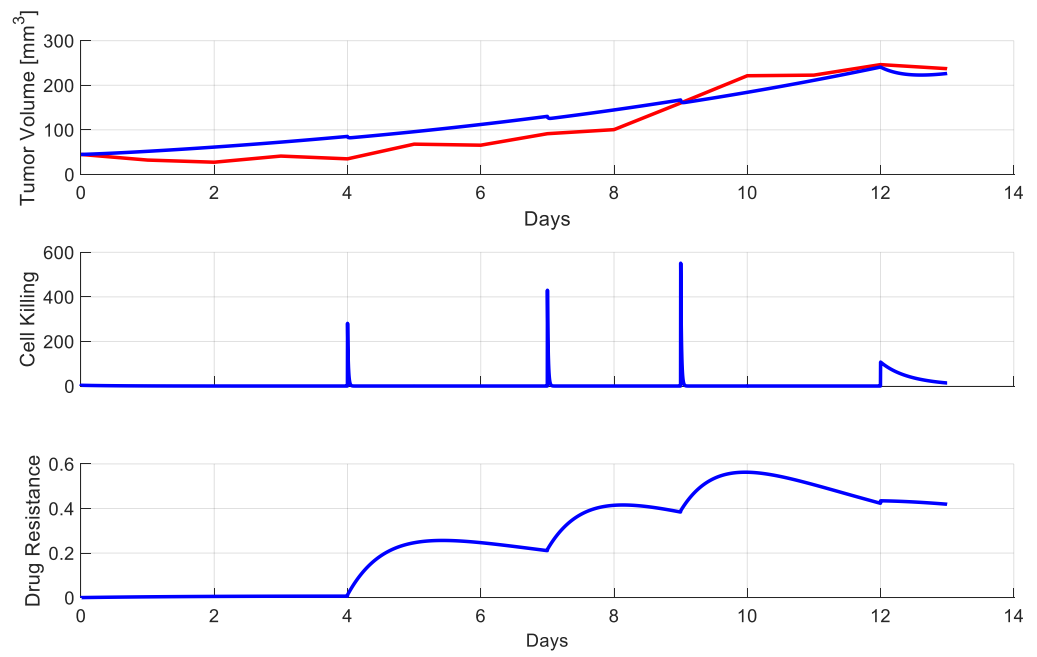

D

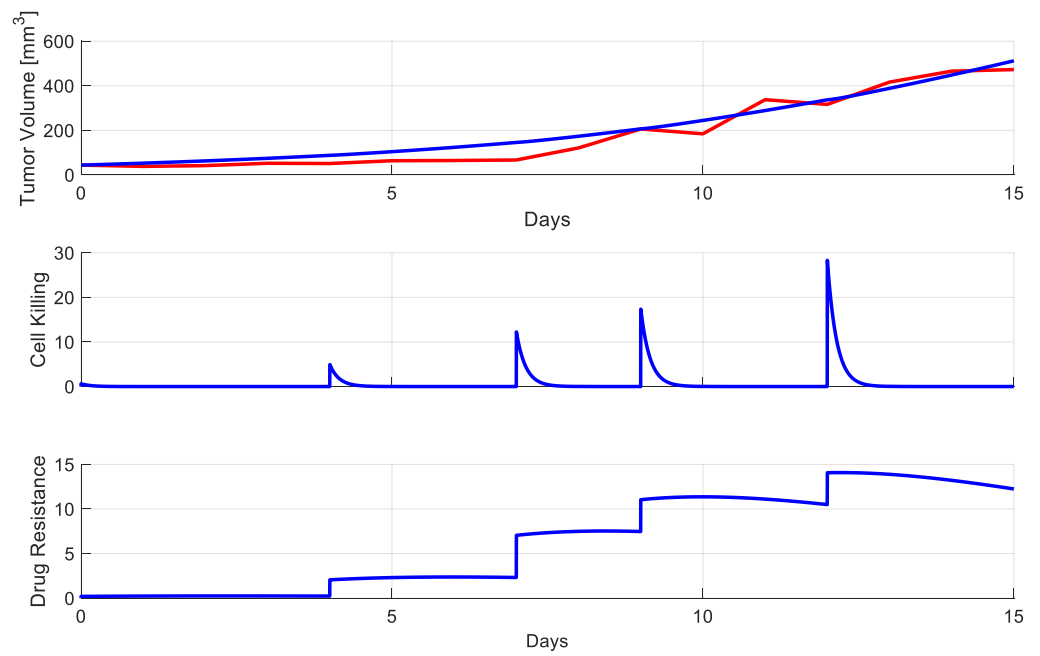

E

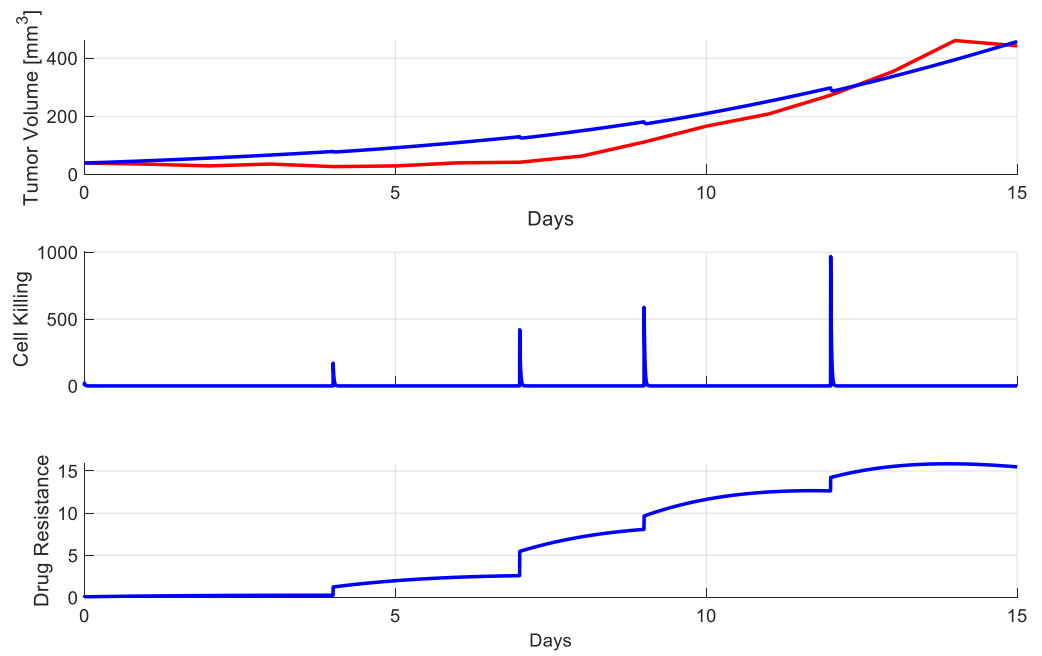

F

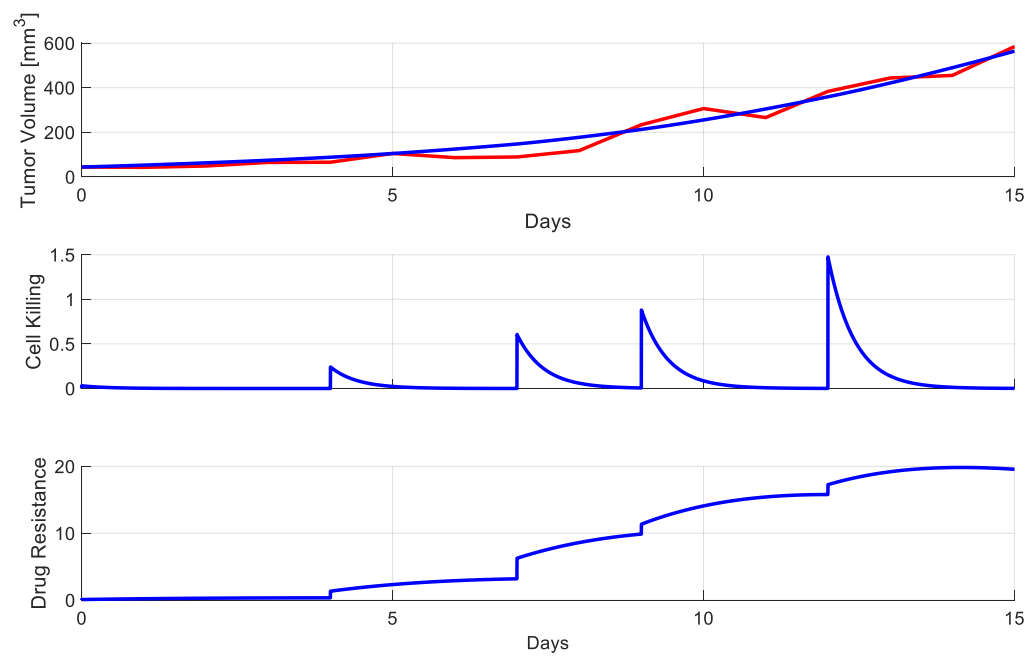

G

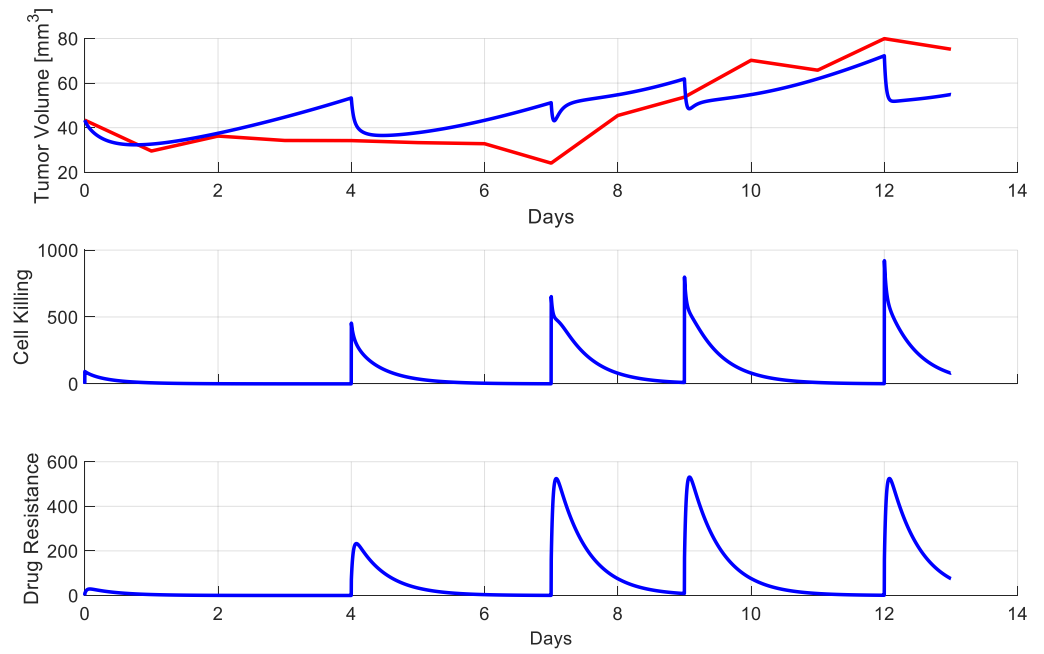

H

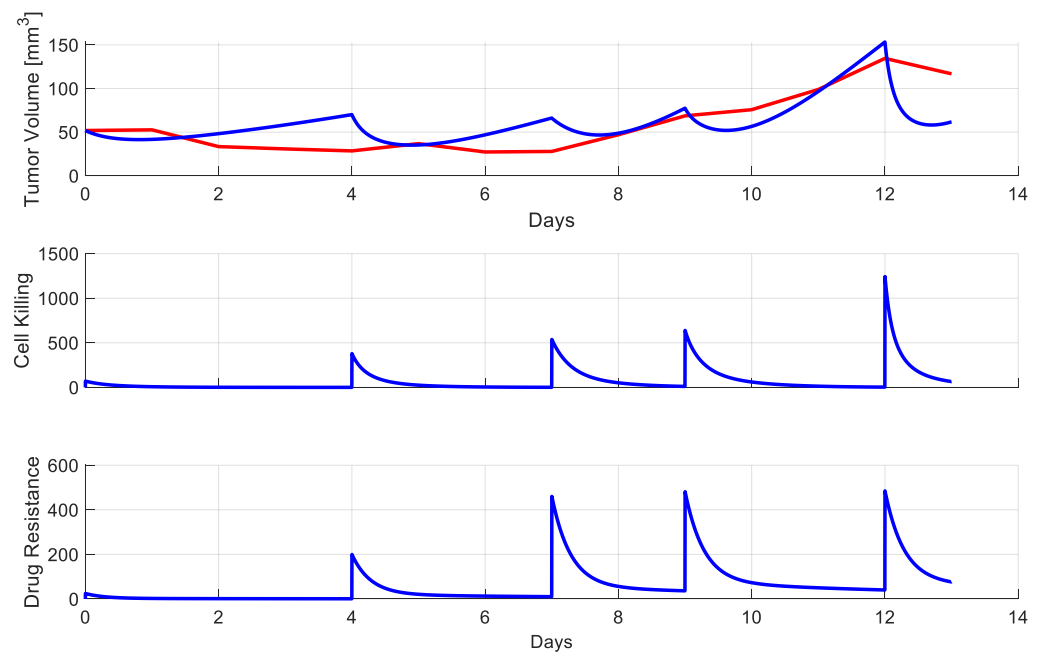

I

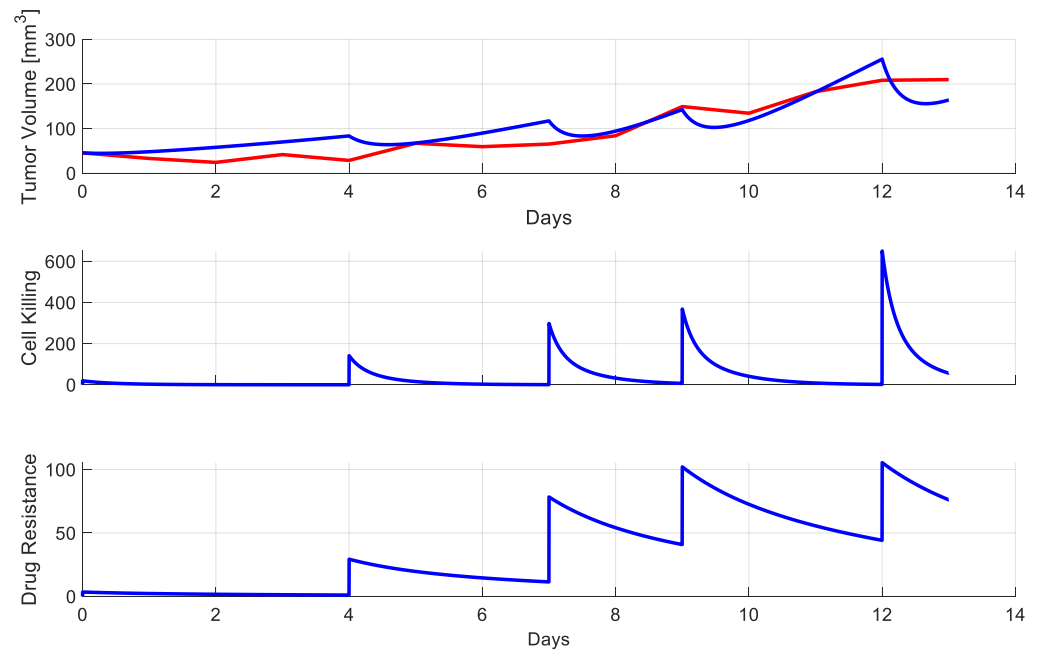

J

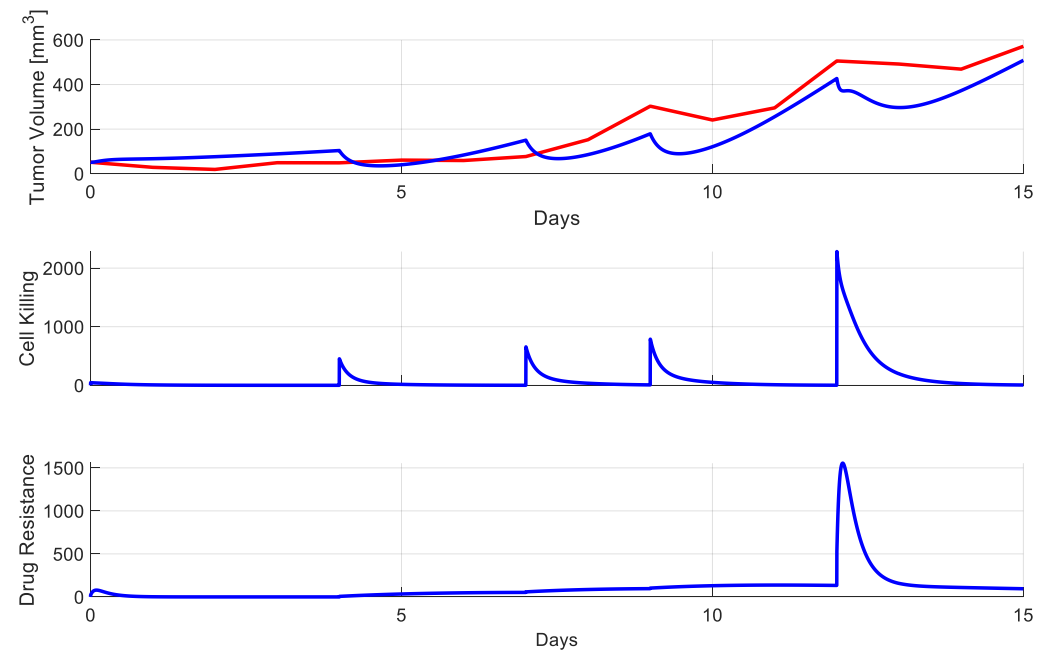

K

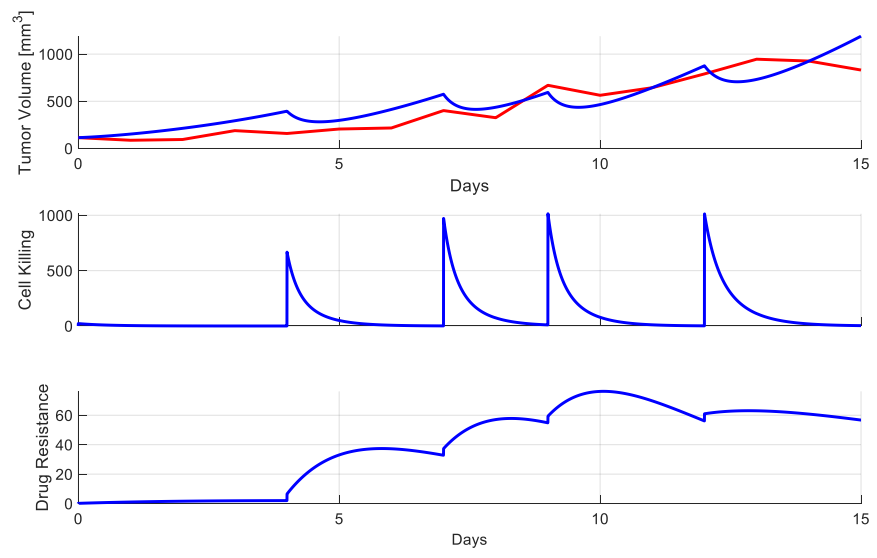

L

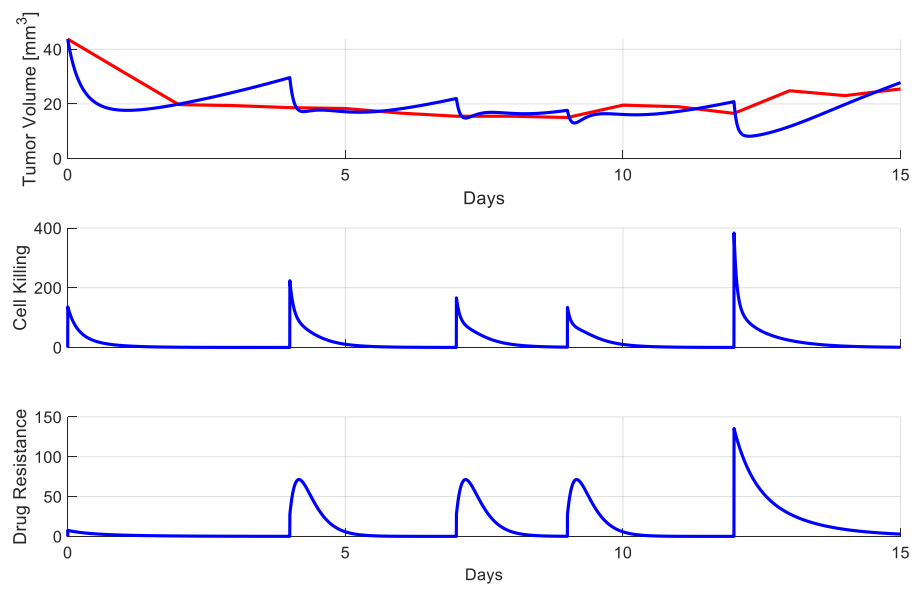

Supplement: Supplementary Figure 2 — Output of the mathematical model (n(t)) and the measured tumor size, CK(u(t)) and R(u(t)) for different animal models. Each graph has three parts: tumor volume, cell killing, and drug resistance. The blue line shows real rate of tumor growth with DAPT treatment to control group in each mice, and the red line is the growth rate of DAPT treatment in each mice that were calcuted with mathematical model. Whatever the lines of the red and blue are more consistent, it means that the model is more similar to reality and more accurate. The total of these 3 parts provide killing factor, which, if the killing factor was negative, the drug would be effective and if the killing factor was positive, treatment would be harmful. (A–L) Animals number 1 to 13. [file Data_Sheet_2.PDF]
