# Supplementary material for: Long-Term Inhibition of Notch in A-375 Melanoma Cells Enhances Tumor Growth Through the Enhancement of AXIN1, CSNK2A3, and CEBPA2 as Intermediate Genes in Wnt and Notch Pathways
Source: Front Oncol. 2020 Jun 30;10:531. doi: 10.3389/fonc.2020.00531 (PMC7338939; doi:10.3389/fonc.2020.00531)
Supplement: Supplementary Table 1 — The sequences of the primers used for sequencing. [file Table_1.docx]

**Supplementary table 1: The sequences of the primers used for sequencing.**

| Gene | Forward primer (5′→3′) | Reverse primer (5′→3′) |
| --- | --- | --- |
| *BRAF*-exon 15 | TCATAATGCTTGCTCTGATAGGA | GGCCAAAAATTTAATCAGTGGA |
| *NRAS*-exon 1 | CAGGTTCTTGCTGGTGTGAAA | CTACCACTGGGCCTCACCTCTATGG |
| *NRAS*-exon 2 | GTTATAGATGGTGAAACCTG | ATACACAGAGGAAGCCTTCG |
| *GAPDH* | CAAGGTCATCCATGACAACTTTG | GTCCACCACCCTGTTGCTGTAG |
